# Difference in Rumor Dissemination and Debunking Before and After the Relaxation of COVID-19 Prevention and Control Measures in China: Infodemiology Study

**DOI:** 10.2196/48564

**Published:** 2024-05-15

**Authors:** Xiaoqi Liu, Qingyuan Hu, Jie Wang, Xusheng Wu, Dehua Hu

**Affiliations:** 1 Department of Biomedical Informatics School of Life Sciences Central South University Changsha, Hunan China; 2 Department of Research The Third Xiangya Hospital of Central South University Changsha, Hunan China; 3 School of Life Sciences Central South University Changsha, Hunan China; 4 Shenzhen Health Care Development Research and Data Management Center Shenzhen, Guangdong China

**Keywords:** new stage, public health emergency, information epidemic, propagation characteristic, debunking mechanism, China

## Abstract

**Background:**

The information epidemic emerged along with the COVID-19 pandemic. While controlling the spread of COVID-19, the secondary harm of epidemic rumors to social order cannot be ignored.

**Objective:**

The objective of this paper was to understand the characteristics of rumor dissemination before and after the pandemic and the corresponding rumor management and debunking mechanisms. This study aimed to provide a theoretical basis and effective methods for relevant departments to establish a sound mechanism for managing network rumors related to public health emergencies such as COVID-19.

**Methods:**

This study collected data sets of epidemic rumors before and after the relaxation of the epidemic prevention and control measures, focusing on large-scale network rumors. Starting from 3 dimensions of rumor content construction, rumor propagation, and rumor-refuting response, the epidemic rumors were subdivided into 7 categories, namely, involved subjects, communication content, emotional expression, communication channels, communication forms, rumor-refuting subjects, and verification sources. Based on this framework, content coding and statistical analysis of epidemic rumors were carried out.

**Results:**

The study found that the rumor information was primarily directed at a clear target audience. The main themes of rumor dissemination were related to the public’s immediate interests in the COVID-19 field, with significant differences in emotional expression and mostly negative emotions. Rumors mostly spread through social media interactions, community dissemination, and circle dissemination, with text content as the main form, but they lack factual evidence. The preferences of debunking subjects showed differences, and the frequent occurrence of rumors reflected the unsmooth channels of debunking. The *χ*^2^ test of data before and after the pandemic showed that the *P* value was less than .05, indicating that the difference in rumor content before and after the pandemic had statistical significance.

**Conclusions:**

This study’s results showed that the themes of rumors during the pandemic are closely related to the immediate interests of the public, and the emotions of the public accelerate the spread of these rumors, which are mostly disseminated through social networks. Therefore, to more effectively prevent and control the spread of rumors during the pandemic and to enhance the capability to respond to public health crises, relevant authorities should strengthen communication with the public, conduct emotional risk assessments, and establish a joint mechanism for debunking rumors.

## Introduction

### Background

Since the outbreak of COVID-19, with the development of information technology and the improvement of digital technology, a large number of epidemic and related information has been fermented and disseminated on various network platforms. The false information has been overwhelming, making it difficult to distinguish truth from falsehood. This phenomenon has also been referred to as an “infodemic” by the World Health Organization [1]. Under the infodemic, rumors such as “Delta and Omicron are co-circulating” and “the State Council Joint Prevention and Control Group is formally removed from its duties” spread widely, and the harm of rumors cannot be underestimated. First of all, such epidemic-related rumors not only lead to the adoption of excessive or incorrect epidemic prevention measures by the public but also easily undermine the public’s confidence in epidemic prevention, causing secondary harm to social order. In addition, with rumors abound, scientific voices will be drowned out. As the infodemic spreads, false information is spread erroneously, whereas scientific, correct, and official epidemic and related information is not properly disseminated due to various rumors. This makes it difficult for some people to discern reliable sources of information from the massive amount of information available, thereby causing social panic and seriously threatening social stability.

The information environment is highly uncertain, and the characteristics of rumor dissemination will also change with different pandemic stages. Therefore, comparing and analyzing the characteristics of rumor dissemination before and after the new stage of epidemic prevention and control, as well as exploring the debunking mechanism under the new stage of epidemic prevention and control, is of great significance for the governance of rumors, for relevant departments to carry out debunking work, and to avoid public health crises. This study will comprehensively analyze large-scale network rumors related to the epidemic before and after the release of the epidemic prevention and control measures and explore them from multiple dimensions, such as the construction of rumor content, rumor dissemination, and debunking response. Based on this, the characteristics of the dissemination of COVID-19 network rumors before and after the release of the epidemic prevention and control measures and the debunking mechanism will be proposed, providing a reference for the governance of network rumors of sudden public health events by relevant departments and the development of debunking work under the information epidemic.

### Conceptual Definition and Related Research

#### Definition and Characteristics of Rumors

The definition of “rumor” has evolved over time, and the “rumor” initially mentioned in “Eastern Zhou States” refers to false information intentionally fabricated without a factual basis [2]. Further research introduced various definitions across disciplines. In communication studies, rumors are information lacking a factual basis, potentially including defamatory elements [3]. Knapp [4] describes rumors as declarations that are meant to be believed, related to current events, and spread without official confirmation. In social psychology, rumors are defined as baseless content spread by individuals or groups with specific motives [5]. Sun [6] views rumors as propositions related to current events, spread mainly through oral communication, but without a solid basis for their accuracy. Rumors are fundamentally unverified and lack evidence. The rise of social media has brought concepts such as “misinformation,” “disinformation,” and “fake news” to the forefront. “Misinformation” refers to inadvertently shared false information, whereas “disinformation” is the deliberate spread of falsehoods [7]. “Fake news” is defined as falsehoods designed to deceive by mimicking real news [8], with Tandoc [9] categorizing fake news into 6 types, including news satire and parody. Network rumors emerge with internet technology, differing from traditional rumors in spread speed, range, and methods. Defined as rumors spread through the internet without deviating from the basic characteristics of traditional rumors [10], they play a crucial role in the generation, dissemination, and impact of rumors on the internet [11]. Compared to traditional rumors, network rumors feature rapid spread, wide reach, and high influence and are challenging to control effectively [12,13].

#### The Process and Patterns of Rumor Propagation

Studying how rumors spread can help curb the impact of destructive misinformation on the internet [14]. Research into rumor spreading dates back to Allport and Postman [15], who introduced the rumor formula as a function of importance and ambiguity in their book *The Psychology of Rumors*. Recent studies have explored rumor spreading patterns, with Zhang et al [16] identifying epidemic-related, web-based rumors as community, micro, and circle spreading, and Tong and Yu [17] summarizing web-based rumor spreading patterns as 1-way linear, multidirectional network, and umbrella-shaped propagation. Simulation models for analyzing rumor content have also advanced. For instance, Xi and Yong [18] proposed a model for public opinion dissemination on social networks, Han et al [19] analyzed network topology in rumor spreading, Wu et al [20] developed a rumor-spreading model based on recommendation systems, and Fang and Yang [21] studied rumor spreading and refutation effectiveness on Weibo under real-name systems. Moreover, epidemic models have been applied to social network rumor propagation studies, based on similarities between rumor spread and disease transmission mechanisms [22], including SI (susceptible and infectious) [23-25], SIS (susceptible, infectious, and susceptible) [26-28], SIR (susceptible, infectious, and recovered) [29-31], and SEIR (susceptible, exposed, infectious, recovered) [32-34] models, representing susceptible, infected, recovered, and exposed states, respectively [35]. Zhu et al [36] introduced a SIS model with a forced silence function, Sun et al [37] applied uncertain differential equations to study an SIR model, Hosseini and Zandvakili [38] added rumor delay and countermeasure mechanisms to an SIR model, and Chen et al [39] proposed a new SEIOR (ignorant, hesitators, spreaders, rumor debunkers, and immunizers) model incorporating debunkers.

#### Governance and Refutation Mechanisms of Rumors

Rumor governance involves 5 main mechanisms: web-based rumor warning and monitoring, improving information disclosure, postevent evaluation, legal mechanisms for web-based rumor punishment, and long-term education [40]. Effective curbing of fake news spread can be achieved through enhancing individual abilities to recognize fake news and structural changes in platform policies [41]. Media literacy training is suggested to prevent misinformation uptake, with a recommendation for tools to identify harmful misinformation [42]. The refutation mechanism, addressing rumors that have already occurred, includes top-down and bottom-up approaches. The top-down approach involves proactive dissemination of official information, whereas the bottom-up approach refers to seeking expert verification of public feedback or rumors and providing feedback through refutation articles [43]. Governments are advised to release official rumor refutation information promptly and ensure its continuous dissemination [44]. Establishing rumor monitoring and refutation mechanisms on various platforms, making policy adjustments according to different stages, and timely releasing official information are recommended [45]. Active dissemination of official information and public engagement in verification can effectively eliminate false information spread.

## Methods

### Sampling and Sample

This study focused on web-based rumors and used rumors related to the COVID-19 epidemic as the research sample. Since rumors originate from various social media platforms such as WeChat, Weibo, Douyin, Kuaishou, and Toutiao, during the epidemic, various media outlets have also launched various epidemic information refutation zones, such as the Hubei Network Refutation WeChat public account, Toutiao Refutation, and Gansu Network Refutation Reporting Platform, etc. Refutation information is relatively scattered. In comparison, the China Internet Joint Rumor Refutation Platform is sponsored by the Central Cyberspace Affairs Commission’s Illegal and Harmful Information Reporting Center. Hosted by Xinhua Net, it has the function of reporting and verifying rumors and can obtain authoritative refutation information from relevant departments and experts. Therefore, the rumor samples in this study are more representative. In addition, since the new stage of epidemic prevention and control has just begun, the number of rumor samples is relatively small. Therefore, we decided to conduct further searches on Toutiao to expand the sample size.

In this study, web crawling tools were initially used to crawl all entries to date (March 2, 2023) from the “Epidemic Prevention and Control Refutation Zone” of the China Internet Joint Rumor Refutation Platform. Subsequently, searches were conducted on Toutiao using the keyword “epidemic debunking” for each of the 31 provinces (direct-controlled municipalities and autonomous regions), and entries from the “News” section were collected. The samples from both platforms were then consolidated, using a fuzzy matching method based on title fields to eliminate duplicate rumor samples while excluding rumors already confirmed as facts. In the end, a total of 672 rumors were obtained as the final research sample. The timeframe of the rumor samples spanned from April 19, 2022, to March 2, 2023, covering both the period before the relaxation of epidemic measures (from April 19, 2022, to November 30, 2022) and the early stage of the new phase of epidemic prevention and control (from December 1, 2022, to March 2, 2023).

### Coding Category Construction

Combining with the Laswell 5W communication model [46], the content to be studied in this paper can be roughly divided into “communicators,” “content,” “channels,” “audiences,” and “effects.” However, based on the content of the selected web-based rumors and considering the complexity and operability of the analysis of “audiences” and “effects,” the categories of this study were composed of “involved entity,” “propagation content,” “emotional expression,” “propagation channel,” “propagation form,” “debunking entity,” and “verification source” [47]. The specific category analysis framework is shown in the Table 1.

**Table 1:** Analysis framework and category construction.

Analytical and statistical categories		Category description	Observation pathway
**Content construction**			
	Involved entity	Entities involved in the spread of rumors	Virus Patients Transportation department Enterprises Schools Hospitals Government agencies Others
	Propagation content	Division of the themes of rumor content	Epidemic situation Epidemic anecdotes Epidemic prevention knowledge Epidemic prevention measures Others
	Emotional expression	Emotional tendencies conveyed in rumor content	Fear Worry Sadness Anger Doubt Recognition Opposition Surprise No clear emotion
**Rumor dissemination**			
	Propagation channel	Platforms and methods of rumor dissemination	Douyin Toutiao WeChat groups or Moments Weibo Others or unknown
	Propagation form	Modes of presenting rumor content	Images Text Video Others or unknown
**Refutation and response**			
	Debunking entity	Publisher of refutation information	Media Government Refutation platforms Collaboration
	Verification source	Sources used for verifying rumor information	Relevant government departments Authoritative experts Enterprises Others or unknown

During the COVID-19 epidemic, the entities involved were complex and diverse. Rumors involving multiple entities were classified based on the most important entity. The specific categories of the content are shown in Table 2. The epidemic situation included information directly related to the occurrence and development of the epidemic, such as epidemic data, trends, regions, policies, and other information. Epidemic curiosities included all kinds of strange stories related to the epidemic, such as epidemic events, aid, heroes, and other information. Epidemic prevention knowledge included related knowledge on interpreting and coping with the epidemic, such as epidemic prevention and control, transmission, causes, science popularization, and other information. Epidemic prevention measures included the epidemic prevention and control measures taken by relevant departments, such as epidemic detection, isolation, treatment, and other information. Anything that did not fall into these 4 categories was classified as other.

**Table 2:** Subcategories of rumor content in epidemic spread.

Subcategory	Rumor content
Epidemic situation	Epidemic data: including statistics such as the number of infections, number of recoveries, number of deaths, etc Epidemic trend: including predictions on the development, changes, and direction of the epidemic Epidemic region: including information on epidemic areas, severity, risk levels, etc Epidemic policies: including epidemic-related policies and measures issued by various levels of government
Epidemic oddities	Epidemic events: including various events and accidents related to the epidemic Epidemic aid: including information on domestic and foreign aid, donations, etc Epidemic heroes: including the deeds of frontline workers such as medical staff and volunteers
Epidemic prevention knowledge	Epidemic prevention and control: including preventive measures, personal protective knowledge, etc Epidemic transmission: including information on transmission routes, transmission methods, etc Epidemic causes: including relevant knowledge on virus sources, infection mechanisms, etc Epidemic science popularization: including various popular science knowledge, epidemic interpretation, scientific research results, etc
Epidemic prevention measures	Epidemic detection: including virus detection, nucleic acid detection, and other related information Epidemic isolation: including home isolation, centralized isolation, and other epidemic prevention measures Epidemic treatment: including various treatment plans, drugs, vaccines, and other information Epidemic resumption: including information on resumption plans and measures of enterprises, schools, and other units

Emotional expression is the emotional tendency of rumor information presentation. Due to the extremely rich types of emotions and the fact that the same rumor may express multiple emotions, to facilitate statistical classification, this paper defined a rumor’s emotional expression category based on the most obvious emotional tendency in the rumor information. The specific subcategories of emotional expression are shown in Table 3. The emotional expression of rumors can be divided into negative emotions, positive emotions, and no obvious emotions. Among them, negative emotions included fear, doubt, worry, opposition, anger, and sadness, and positive emotions included approval [48]. As surprise can represent both positive and negative emotions in different contexts, it was not classified into emotional attributes here.

**Table 3:** Subcategories of emotions expressed in COVID-19 rumors.

Emotion subcategory	Description
Fear	Focuses on information related to the spread of the virus and the death rate, which can cause panic and fear among people
Worry	Focuses on the impact of the pandemic on individuals, families, and society, which can cause people to worry and feel anxious
Sadness	Focuses on information and reports related to victims of the pandemic, which can lead to feelings of sadness
Anger	Focuses on information related to the origin of the virus and the handling of the pandemic, which can cause people to feel dissatisfied and angry
Doubt	Focuses on information related to the authenticity of pandemic data and the credibility of government agencies, which can cause people to be suspicious and doubtful
Approval	Focuses on information related to known methods of prevention and scientific research findings, which can cause people to accept and approve of such information
Opposition	Focuses on voices of opposition to pandemic response measures, which can cause people to feel resentment and dissatisfaction
Surprise	Focuses on strange and unusual events related to the pandemic and heroic deeds of medical workers, which can cause people to feel surprised and moved
Not apparent	Focuses on other information related to the pandemic that may not have an obvious emotional tendency

Rumors often spread through multiple media platforms. Based on content analysis of rumor samples, this paper classified the channels of rumor dissemination into 5 main forms listed in Table 1. The propagation form referred to the way the rumor information was presented, mainly divided into 3 categories: images, text, and video. The debunking entity referred to the entity that clarified the rumor, mainly including media, government, and debunking platforms. The verification source referred to authoritative sources of information used to verify the rumor. In the context of epidemic rumors, verification primarily came from relevant government departments, authoritative experts, and enterprises, which are the parties referred to in the rumor information or authoritative entities in the relevant field.

In addition, in the coding process, it was necessary to scientifically design and control the coding process to ensure the reliability of the coding. The coding reliability assurance measures in this study were as follows: (1) three coders with qualitative research and coding experience encoded the analysis text and conducted a Krippendorff α coefficient test [49]; (2) all personnel received coding training, selected some indicators at random for trial coding, discussed the coding results, improved coding standards, and reached a consensus for formal coding; and (3) during the formal coding, coders independently completed the coding work, communicated the coding results together after the completion of the coding work, and discussed the points of disagreement to ensure the coding reliability. By sorting out the data of coders and using SPSS statistical software (version 25.0; IBM Corp) to conduct a Krippendorff α reliability test on the data, the results showed that the reliability coefficients were all greater than 0.80, demonstrating a high level of reliability.

### Statistical Analysis

Data were extracted from the “Epidemic Prevention and Control Rumor Refutation Special Area” of the China Internet Joint Rumor Refutation Platform and Toutiao. The extracted data were organized according to the analysis framework and category construction of this study, processed using Microsoft Excel, and analyzed using SPSS statistical software. The *χ*^2^ test was used for categorical data, and the Pearson *χ*^2^ test or likelihood ratio *χ*^2^ test was selected based on the sample size and whether the theoretical frequency in each cell was greater than 5. A difference was considered statistically significant at *P*<.05.

### Ethical Considerations

The data mentioned above are publicly accessible, and none of the data were used to trace personal identification details such as ID numbers, birth dates, or IP addresses. The authors sought advice from the institutional review board of the respective institution and were informed that no approvals were required.

## Results

### Content Construction: Features of Entities, Content, and Emotional Expression

#### Involved Entity: The Vast Majority of Rumor Information Dissemination Entities Were Relatively Clear

Regarding the entities involved in rumor content, the relevant entities that society was widely concerned about such as viruses, patients, transportation departments, enterprises, schools, hospitals, and government agencies were mentioned in the rumors during the epidemic period. Comparing the keyword clouds of rumor dissemination before and after the epidemic outbreak (Multimedia Appendix 1), rumors in both periods mainly targeted “nucleic acid testing,” “hospitals,” “patients,” and other entities. However, after the epidemic was lifted, rumors related to “virus” and “vaccines” increased relatively, which may have been caused by the adjustment of epidemic prevention and control policies and the public’s panic about the uncertain direction of virus mutation. Among them, “virus” and “patients” had a strong correlation, and the rumors related to “virus” mainly focused on its development status and prevention and treatment, such as “COVID-19 is not a severe flu, it is a minor AIDS,” “Delta and other variants are circulating in Beijing’s northern region,” and “Delta and Omicron are co-circulating and may recombine.” Rumors related to “patients” were closely related to the occurrence and development process of COVID-19, including prevention, treatment, rehabilitation, and various stages of patient symptoms and behavioral dynamics: for example, “A COVID-19 positive patient in Dadixiang Township died after taking a bath during the recovery period” and “Many people who relapsed were infected again because they forgot to change their toothbrush after their test results turned negative.” Such rumors make it difficult for the public to distinguish between true and false information, causing great panic in society.

#### Content Dissemination: The Content Themes Mainly Focused on the Areas Closely Related to the Public’s Vital Interests in the Context of the Epidemic

Analysis of the content themes of rumor samples showed that during the COVID-19 pandemic, rumors can be mainly categorized into 4 types: the pandemic situation, strange occurrences related to the pandemic, knowledge about preventing the spread of the virus, and measures to prevent the spread of the virus. Among them, the pandemic prevention and control measures were the most frequent topic, accounting for 78.3% (526/672) of all content, as shown in Table 4. The difference in content themes between the pre- and postlockdown periods of the pandemic was statistically significant (*χ*^2^_4_=29.374, *P*<.001). Comparing the distribution of content themes before and after the lockdown period (Multimedia Appendix 2), it was found that rumors about measures to prevent the spread of the virus dominated in both periods. However, during the high-intensity outbreak period, there was a relative increase in rumors about knowledge related to prevention and a decrease in rumors about pandemic prevention and control measures. This indicated that in situations where the prevention and control mechanism changed and the uncertainty of the pandemic increased, people were more inclined to obtain information about preventing the virus rather than general information about the pandemic. In addition, it was observed that the specific content of rumors was concentrated in areas of public interest during the COVID-19 pandemic, such as silent management during the pandemic, city lockdown, traffic control, and delayed resumption of work and school. Such rumors were particularly prominent. COVID-19–related rumors also took various forms, including fabrication, tampering, and misinterpretation, and were generated for various reasons, such as to attract attention, hostility, or subjective conjecture.

**Table 4:** Overall distribution of epidemic rumors.

Content theme	Rumors (N=672), n (%)
Epidemic situation	133 (19.8)
Epidemic oddities	114 (17)
Epidemic prevention knowledge	56 (8.3)
Epidemic prevention measures	279 (41.5)
Others	90 (13.4)

#### Emotional Expression: Rumors About the Pandemic Showed Significant Differences in Emotional Expression, With Many Trending Toward Negative Emotions

**Figure 1:**
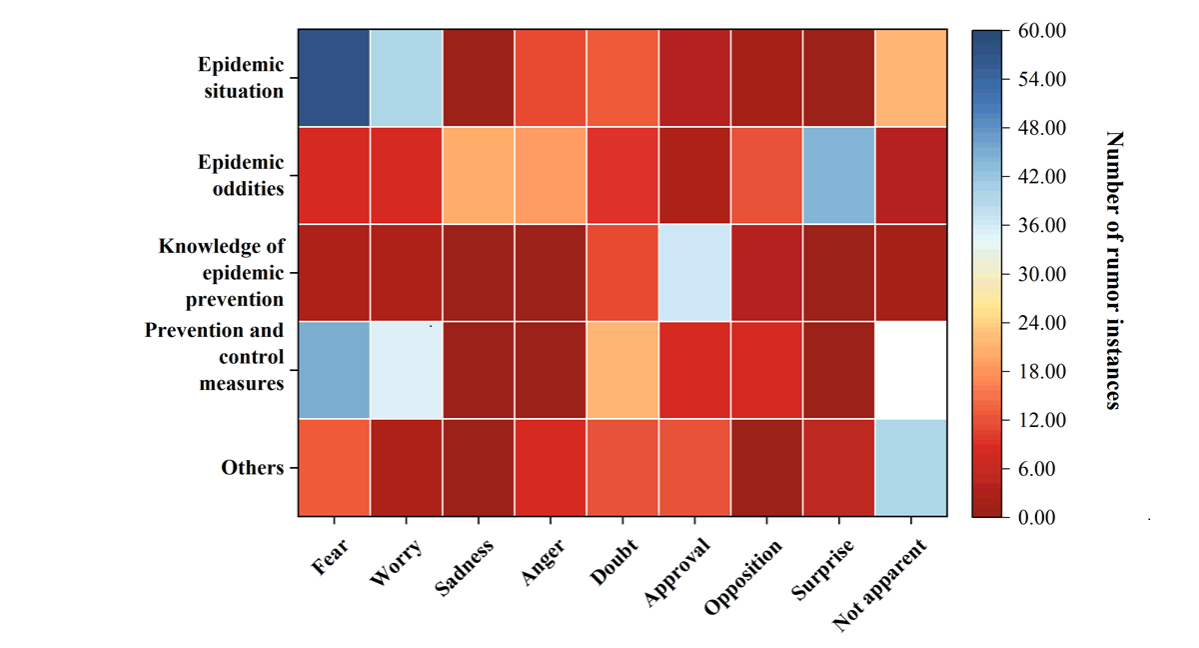
Heatmap of overall sentiment expression in COVID-19 rumor content.

**Figure 2:**
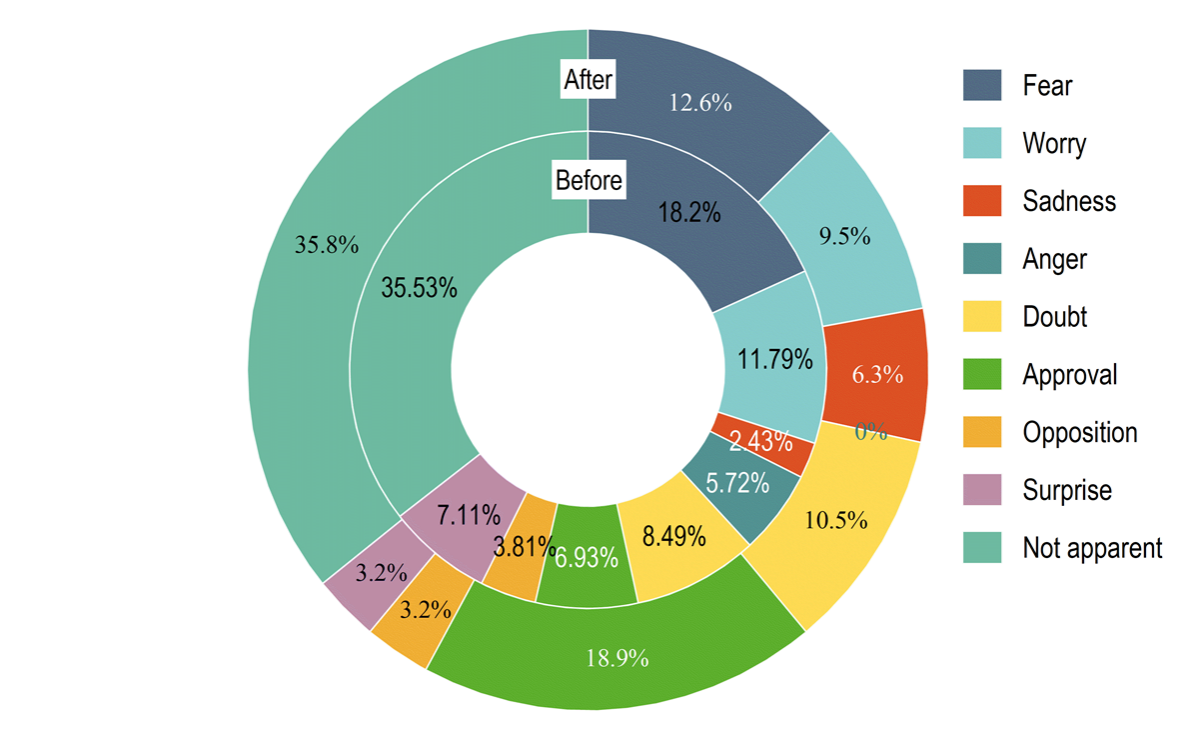
Comparison of emotional expressions in rumors before and after the relaxation of the epidemic prevention and control measures.

Different themes of COVID-19 rumors had distinct emotional preferences (Figure 1). Rumors related to the pandemic situation tended to express fear and concern, whereas those related to bizarre incidents tended to express surprise. Rumors related to knowledge and measures of epidemic prevention tended to express approval, whereas those related to epidemic prevention measures not only showed a tendency toward fear and concern but also aimed to satisfy the public’s need for information without any clear emotional expression. The difference in emotional expression between rumors before and after the pandemic was statistically significant (*χ*^2^_8_=28.924, *P*<.001). Comparing the emotional expressions of rumors before and after the pandemic (Figure 2), it was observed that during the initial stage of the new epidemic prevention and control period, the proportion of fear, concern, anger, and surprise expressed in pandemic rumors decreased, whereas the proportion of sadness and questioning increased substantially. At the same time, the proportion of rumors that expressed approval also increased substantially. Moreover, both before and after the pandemic, pandemic rumors tended to express negative emotions (291/577, 50.4% and 40/95, 42%, respectively). More than half of the negative emotions tended to arouse the fear and concern of the public (173/291, 59.4% and 21/40, 52%, respectively), such as “asymptomatic infections will cause a new outbreak of the pandemic.” Such rumors often quickly triggered the fear of the public about the severity of the pandemic, and in an environment where everyone was at risk, people tended to trust them without much discernment. In contrast, rumors related to epidemic prevention knowledge mainly expressed positive emotions and idealized “wishes” for virus prevention and treatment, with the proportion of approval emotions accounting for nearly two-thirds of rumors (20/33, 61% and 13/23, 57%, respectively), such as “drinking garlic water can cure COVID-19, so we should drink more garlic water.” Such rumors often contained positive action hints.

### Process of Rumor Transmission: Rumors Were Mainly Spread Through Social Media Interactions and Community Transmission but Lacked a Factual Basis in the Content

**Figure 3:**
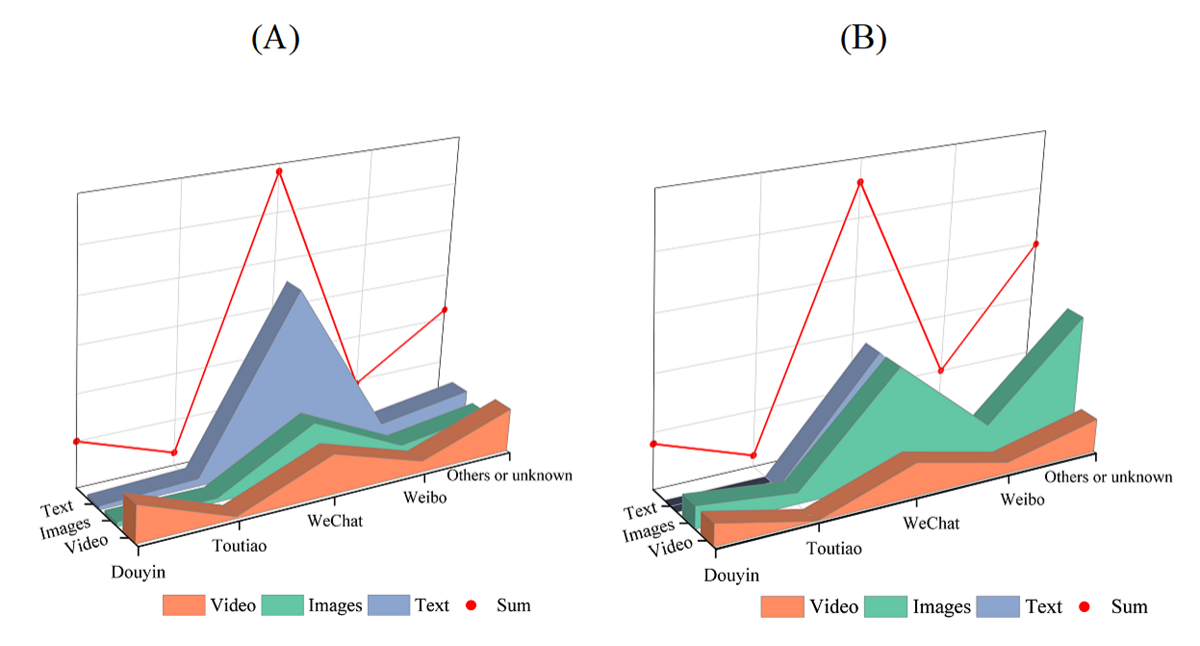
Distribution of channels and forms of rumor dissemination (A) before and (B) after the release of epidemic control measures.

The spread of COVID-19 rumors mainly relied on various social media platforms, and after excluding rumor samples with unknown dissemination channels, it was found that most COVID-19 rumors (306/672, 45.5%) before and after the lifting of restrictions were spread through WeChat groups and Moments (Figure 3). The differences in the dissemination channels and forms of rumors between the 2 periods were statistically significant (*χ*^2^_5_=15.498, *P*=.01; *χ*^2^_3_=33.510, *P*<.001). Among the known forms of rumor samples, text- and image-based rumors were more common before and after the lifting of restrictions. Images and texts are more easily manipulated and distorted compared to videos, making them easier to fabricate. In addition, there were secondary COVID-19 rumors based on images and texts, which were widely spread through social media platforms such as Douyin, Weibo, Toutiao, and Xiaohongshu. At the same time, it is worth noting the phenomenon of using authoritative experts to spread false information on a large scale, such as “The First Hospital of Ningbo warmly reminds that COVID-19 is not an ordinary cold, and each infected person should rest for a month like postpartum women...” There were also a series of rumors fabricated using people’s respect for authoritative experts, such as “The chairman of Changzhou Red Cross claims that drinking water boiled with brown sugar, ginger, scallion, and garlic can almost eliminate the chance of virus infection.” These types of rumors are often titled with eye-catching headlines such as “Emergency Notification from the Community” or “Important Reminder” or were presented in the tone of official announcements such as “Notice from the Joint Prevention and Control Office of COVID-19 in the Community” or “Morning Assessment by the CDC.” They also included phrases such as “Just informing your relatives and family members” or “Forward it to the people you care about” to facilitate circulation and expand the range of dissemination through social relationships.

### Response to Rumors: Different Rumor-Refuting Entities Had Content Preference Differences, and Frequent Rumors Reflected Poor Unobstructed Channels for Rumor Clarification

**Figure 4:**
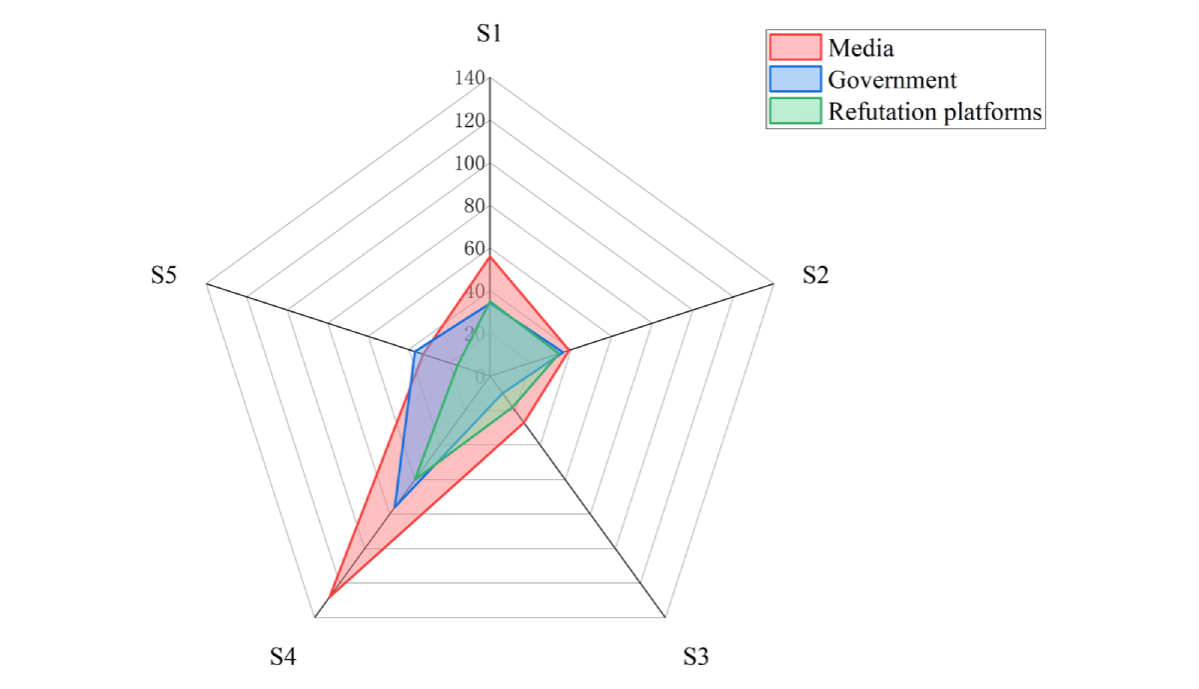
Radar chart of rumor-debunking entities and content categories. The 5 rumor content categories are S1: epidemic situation, S2: epidemic oddities, S3: epidemic oddities, S4: epidemic prevention measures, and S5: others.

The main entities involved in debunking rumors were the media, government, and rumor-refuting platforms, among which the media played a leading role both before and after the release of pandemic control measures (Multimedia Appendix 3), followed by the government and rumor-refuting platforms. The difference in rumor debunking between the 2 periods (before and after the lifting of pandemic control measures) was statistically significant (*χ*^2^_3_=13.894, *P*=.003). By comparing the debunking entities and the content of rumor dissemination (Figure 4), it was found that all 3 entities tended to focus on rumors related to epidemic prevention measures. In addition, compared with the government and rumor-refuting platforms, the media was the main force in addressing and debunking rumors related to epidemic prevention measures, the epidemic situation, and epidemic prevention knowledge. The government, as the overall manager of epidemic prevention and control, can effectively intervene in epidemic-related rumors at the macro level and stabilize social emotions. The media can collaborate with official authorities to obtain firsthand information on epidemic developments, such as virus transmission, confirmed patients, and quarantine treatment, while also meeting the public’s need for epidemic prevention information. In addition, tracing and verifying rumors by contacting relevant departments or individuals is currently the main method of rumor management and response. The National Health Commission, Centers for Disease Control and Prevention, and transportation departments, as well as authoritative experts, are the main channels for verification, along with enterprises, schools, hospitals, and individuals. Although the intervention of debunking entities and the use of debunking channels to verify rumors can effectively curb the spread of rumors, the emergence of old rumors in new forms also reflects the phenomenon of unsmooth debunking channels to a certain extent.

## Discussion

### Principal Findings

This paper discussed the characteristics and refutation mechanisms of COVID-19–related, web-based rumors before and after the lifting of pandemic restrictions, covering aspects such as rumor content construction, dissemination, and refutation responses. The study found that most rumors clearly targeted specific entities, with content themes closely related to the public’s immediate interests in the pandemic domain. Rumor dissemination often showed significant emotional expression differences, which were predominantly negative. Rumors were mainly spread through social media interactions within communities and circles, with the text lacking a factual basis. Additionally, the entities refuting rumors showed content preference differences, indicating inefficiencies in rumor-refuting channels.

### Interpretation of Findings

The content themes of epidemic rumors focused on closely related areas of public interest. Relevant departments should attach importance to the public’s interests and adopt targeted measures to prevent rumors. First, due to the lack of understanding of the epidemic situation, some members of the public are prone to fear, which leads to the spread of false information about the epidemic situation, thus causing damage to social order and instability in the social environment [50,51]. Second, due to differences in personal information literacy and unsmooth information communication channels [52], some members of the public are prone to listen to and believe in certain “folk remedies” for COVID-19 and even regard some fake experts’ false epidemic prevention knowledge as a guiding principle. Some unscrupulous merchants take advantage of the public’s epidemic prevention demands and fears to falsely advertise the efficacy of drugs, leading to shortages of some drugs and making it difficult for those who truly need them to obtain them, thus resulting in market chaos [53]. Therefore, relevant departments should proactively understand and grasp the public’s needs and develop differentiated rumor prevention and control plans. Authoritative departments and senior experts should strengthen communication with the public to reduce information asymmetry that leads to the proliferation of rumors [54]. Meanwhile, official releases and authoritative information exchange should be used to compress the space for the spread of rumors [44].

Public emotions are fuel for the spread of rumors during the COVID-19 outbreak, and it is important to assess the risks of public emotions and guide them through media cooperation [55]. During the outbreak, negative emotions such as panic, doubt, opposition, and concern among the public were widespread, and rumors could easily fuel negative emotions, leading to impulsive and irrational behavior [56]. Relevant departments should establish an effective system for assessing social emotional risks, grasp the psychological trends of the public, and provide psychological counseling to mitigate negative emotions and prevent their spread, thereby avoiding serious social harm. At the same time, it is important to also recognize the impact of rumors on positive emotions and take appropriate measures to prevent them based on different emotional responses. Additionally, media coverage plays an important role in guiding public emotions. On the one hand, media coverage can help the public clarify information, correct information biases, and meet their cognitive needs; on the other hand, it can also become a breeding ground for rumors and an ignition source for public opinion [57]. Therefore, relevant departments should work with the media to effectively guide public opinion, create a positive social atmosphere, interact positively with the public, and promote the smooth implementation of COVID-19 prevention and rumor control work.

The spread of rumors often relies on strong ties in social networks [58]. Therefore, it is necessary to enhance the precision of rumor governance and encourage various social actors to actively participate in rumor control. Social media is a major channel for emotional communication and information sharing among the public, and emotional transmission within interpersonal networks can easily cause group resonance. Social networks with strong ties such as WeChat groups and friend circles have become easy platforms for public opinion fermentation and rumor proliferation. In the prevention and control of rumors, relevant departments should attach importance to community and circle transmissions, closely monitor the trend of public opinion, respond in a timely manner to topics that have aroused public discussion, and conduct proper tracing and verification of information, to timely cut off the spread of rumors and respond to them [59]. At the same time, a multiparty joint rumor control mechanism should be established, and relevant government departments should provide corresponding communication channels and upload and issue relevant information in a timely manner [60]. Authoritative experts should proactively communicate with the public in a timely manner to crush false rumors. Relevant media should also collaborate with authoritative officials, fulfill their social responsibilities, fully play the role of public opinion propaganda, and timely meet the information needs of the public.

### Limitations

However, this study still has certain limitations. On the one hand, with the development of internet technology, the spread and governance of network rumors have become more complex. Future research needs to further consider the characteristics of communication subjects and network users, establish more detailed and comprehensive rumor propagation models, and identify more targeted debunking mechanisms. On the other hand, as the pandemic was still in its early stages, the sample size of postpandemic rumors included in this study was relatively small and static. Therefore, future research can explore ways to incorporate more relevant data to establish a complex and timely dynamic model. Furthermore, despite the call in this paper for relevant authorities to closely monitor the trends in public opinion and actively carry out fact-checking, there is currently a lack of clear standards to determine which information should be subject to verification and which may not require detailed scrutiny. In the future, there is a need for in-depth research to establish clear and practical debunking standards. This includes specifying the types of information more likely to capture public attention, identifying information that may pose threats to public safety, and determining which sources are more likely to disseminate accurate information. This approach aims to enhance the effectiveness of debunking efforts and optimize resource use, ensuring that debunking work is more targeted and efficient.

### Conclusions

As a sudden and serious public health emergency of international concern, the COVID-19 pandemic has caused significant harm to social order. Through an analysis of rumors during the COVID-19 pandemic, it is evident that the themes of these rumors revolve around the immediate interests of the public, requiring targeted measures from relevant authorities. Additionally, recognizing the role of public emotions as an accelerant for rumor dissemination, there is a need for risk assessment and media-driven emotional guidance. Rumors often exploit strong social networks for propagation, emphasizing the necessity to enhance precise rumor governance and encourage diverse societal participation. In conclusion, it is emphasized that authorities should prioritize communication with the public, conduct emotional risk assessments, and establish collaborative rumor control mechanisms. This study provides a theoretical basis and effective approach to enhance debunking mechanisms during public health emergencies.

## Appendix

Multimedia Appendix 1 Word cloud maps of entities involved in rumor propagation (A) before and (B) after the release of epidemic control measures.

Multimedia Appendix 2 Comparison of the distribution of epidemic rumors before and after the relaxation of the epidemic prevention and control measures.

Multimedia Appendix 3 Comparison of rumor-debunking entities before and after the relaxation of the epidemic prevention and control measures.

## Abbreviations

SEIOR: ignorant, hesitators, spreaders, rumor debunkers, and immunizers
SEIR: susceptible, exposed, infectious, and recovered
SI: susceptible and infectious
SIR: susceptible, infectious, and recovered
SIS: susceptible, infectious, and susceptible

## Data Availability

The data sets generated during and analyzed during this study are available from the corresponding author on reasonable request.
