# Supplementary figures and images for: Difference in Rumor Dissemination and Debunking Before and After the Relaxation of COVID-19 Prevention and Control Measures in China: Infodemiology Study
Source: J Med Internet Res. 2024 May 15;26:e48564. doi: 10.2196/48564 (PMC11137433; doi:10.2196/48564)

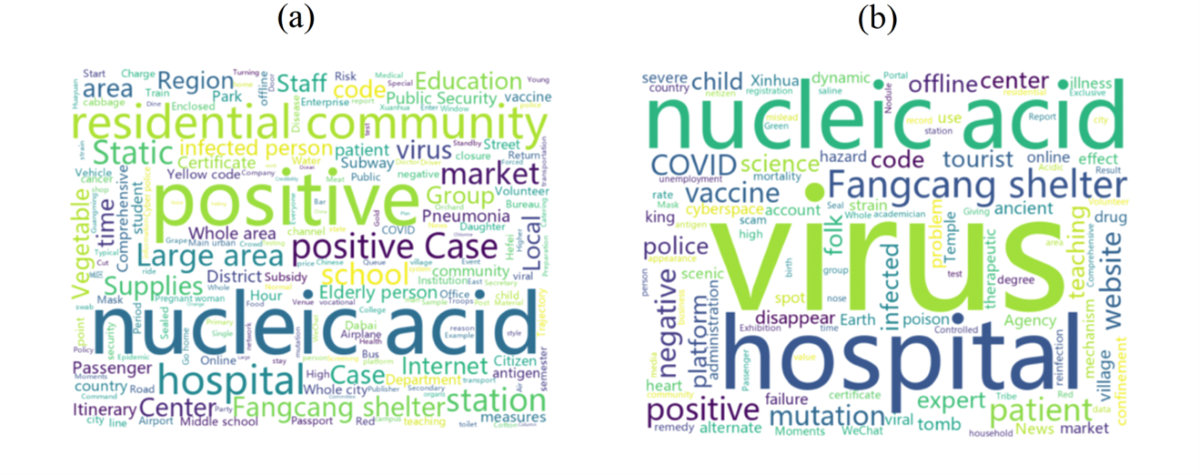

Supplement: Multimedia Appendix 1 [file jmir_v26i1e48564_app1.png]

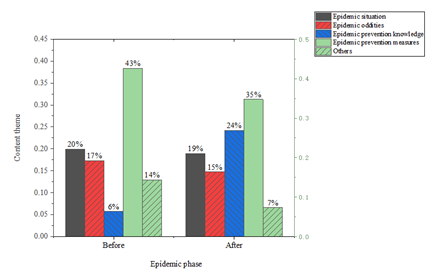

Supplement: Multimedia Appendix 2 [file jmir_v26i1e48564_app2.png]

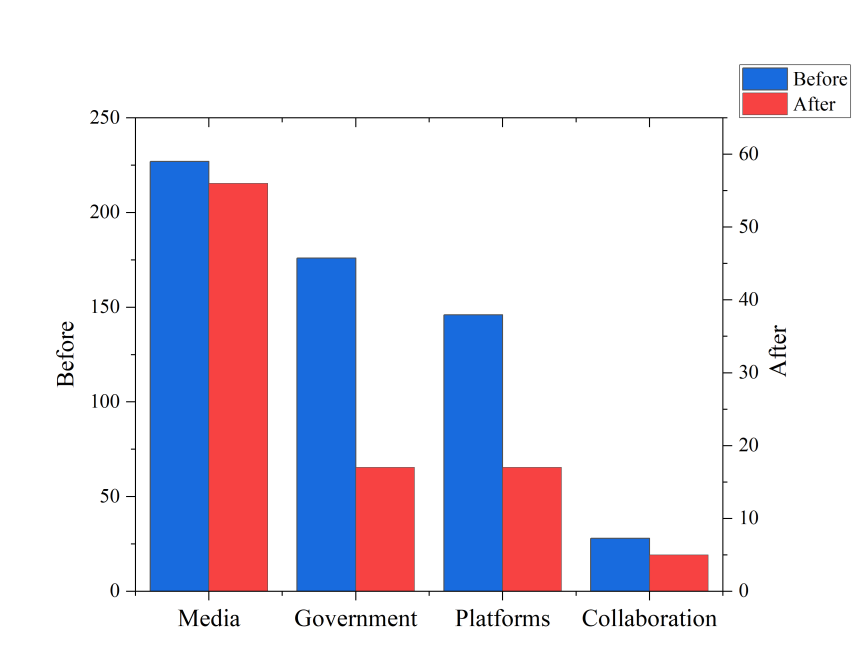

Supplement: Multimedia Appendix 3 [file jmir_v26i1e48564_app3.png]
